# A calcification subtraction method for postmortem coronary computed tomography angiography

**DOI:** 10.1007/s00414-024-03321-0

**Published:** 2024-09-12

**Authors:** Go Inokuchi, Masatoshi Kojima, Fumiko Chiba, Yumi Hoshioka, Maiko Yoshida, Shigeki Tsuneya, Hirotaro Iwase

**Affiliations:** 1 Department of Legal Medicine, Graduate School of Medicine, Chiba University, 1-8-1 Inohana, Chuo-ku, Chiba, 260-8670 Japan; 2 Department of Forensic Medicine, Graduate School of Medicine, The University of Tokyo, 7-3-1 Hongo, Bunkyo- ku, Tokyo, 113-0033 Japan

**Keywords:** Postmortem angiography, Ex situ coronary CT angiography, Coronary subtraction CT angiography, Coronary calcification

## Abstract

Although coronary computed tomography (CT) angiography is a useful tool for evaluating coronary artery lesions both ante- and postmortem, accurate evaluation of the lumen is difficult when highly calcified lesions are present, owing to overestimation of stenosis caused by blooming and partial volume artifacts. In clinical practice, to overcome this diagnostic problem, a subtraction method has been devised to remove calcification by subtracting the precontrast image from the contrast image. In this report, we describe a calcification subtraction method using image analysis software for postmortem coronary CT angiography. This method was devised based on preliminary experimental results showing that the most accurate subtraction was achieved using images reconstructed with a narrower field of view and bone kernel, resulting in higher spatial resolution. This subtraction method allowed evaluation of lumen patency and the degree of stenosis on contrast-enhanced images in a verification using actual specimens where evaluation of the lumen had been difficult because of high calcification. The results were morphologically similar to the macroscopic findings. This method allows more rapid and reliable lesion retrieval and is expected to be useful for postmortem coronary angiography in forensic practice.

## Introduction

Although coronary computed tomography (CT) angiography is useful for evaluation of coronary artery lesions both ante- and postmortem, accurate evaluation of the lumen is difficult when highly calcified lesions are present, owing to overestimation of stenosis caused by blooming and partial volume artifacts. In clinical practice, to overcome this diagnostic problem, a subtraction method has been devised to remove calcification whereby the precontrast image is subtracted from the contrast image [1–7]. The subtraction method does not simply remove the high attenuation areas, including both the calcified area and the surrounding artifact observed on CT, but can also reveal the presence of contrast medium behind the artifact, allowing evaluation of the contrast effect around the calcified area. Since the method was first published [1], there have been scattered reports suggesting that it improves diagnostic performance [2–7]. In histopathological analysis of samples taken at autopsy, the decalcification procedures required for evaluation of calcified lesions are time-consuming and cumbersome. However, if the specimen is cut open without decalcification, the specimen is often destroyed and evaluation of the lumen becomes difficult. Therefore, use of the subtraction technique for postmortem evaluation of the coronary arteries may enable a more rapid and reliable method for retrieval of lesions in addition to the other advantages of using postmortem coronary angiography in forensic practice. Based on the results of a preliminary experiment, we propose use of a novel postmortem coronary CT angiography subtraction method that allows evaluation of severely calcified lesions.

## Materials and methods

In coronary CT angiography subtraction, an image is obtained by registering the precontrast image with the postcontrast image and subtracting the areas recognized as calcification. For registration to be successful, it is important to avoid “misalignment” of the information on precontrast and postcontrast images. Given that the spatial resolution of the image is considered to affect the result of subtraction, we performed a preliminary experiment using a phantom to determine the appropriate imaging conditions.

### Preliminary experiment with a simulated vessel phantom

**Fig. 1:**
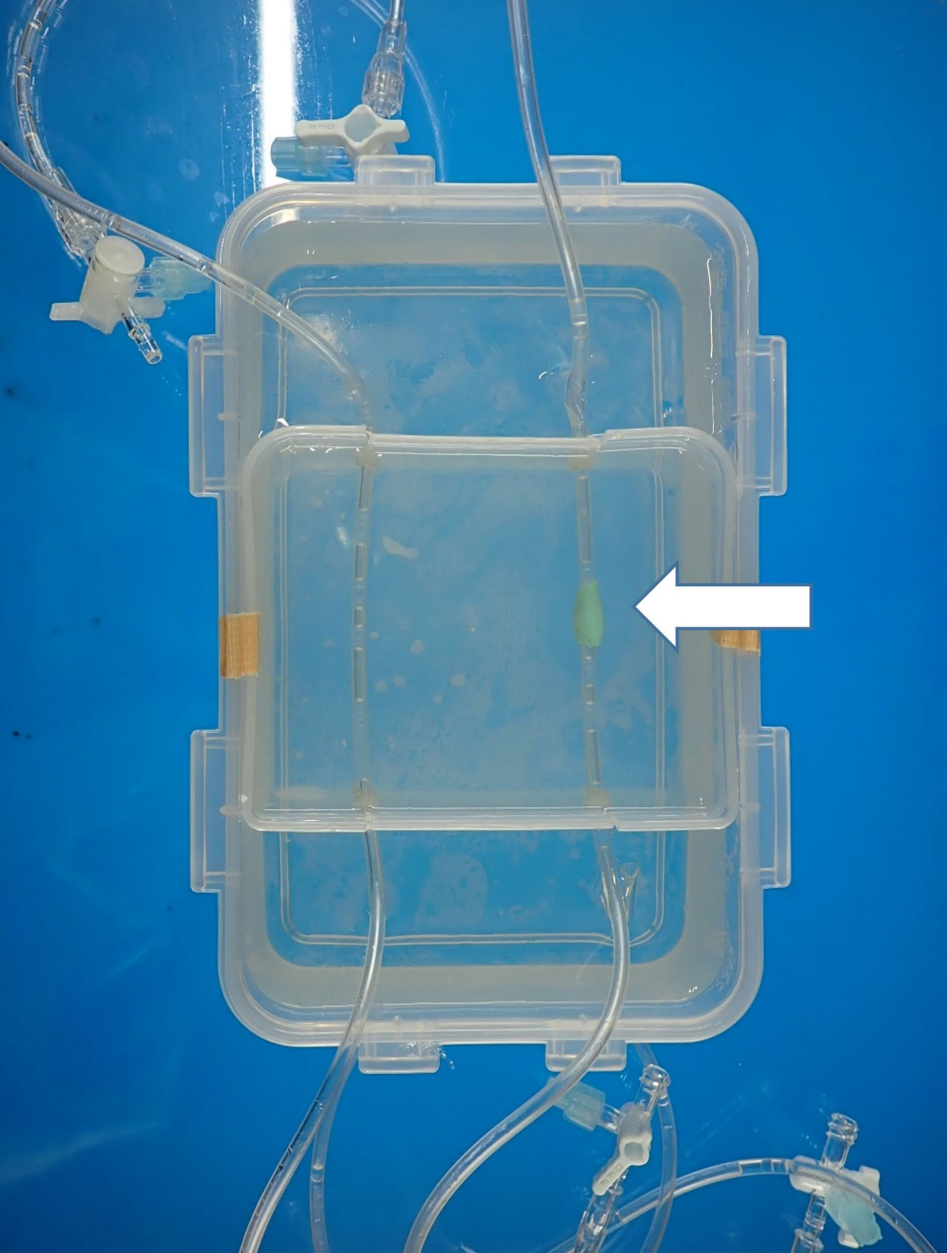
Phantom used in the preliminary experiment. Polyvinyl chloride infusion tubes were used as the simulated vessel and oil clay containing stone powder and calcium carbonate (arrow) as the simulated calcified lesion

Two polyvinyl chloride infusion tubes with an inner diameter of 2.2 mm were prepared as simulated blood vessels. One of the two tubes was coated with oil clay containing stone powder and calcium carbonate to simulated a calcified lesion and fixed in a plastic case. To reduce the artifact caused by the difference in CT values between the simulated vessel and its surrounding area and for stability during scanning, the plastic case was filled with water in which gelatin was dissolved and allowed to cool and solidify (Fig. 1).

### Image acquisition

Using a 64-row multislice CT scanner (Supria Grande^®^ Fujifilm Medical, Tokyo, Japan), two scans of the phantom simulating blood vessels were obtained. One scan was obtained after injection of saline and the other after injection of contrast agent to acquire the raw data set. The following settings were used for acquisition of the raw data: voltage 120 kV, current 75 mA, and collimation 0.63 × 32 mm, rotation time 0.75 and helical pitch 0.5938 for the non-contrast images and voltage 120 kV, current 150 mA, and collimation 0.63 × 32 mm, rotation time 0.75 and helical pitch 0.5938 for the contrast images. The contrast agent used was oil-based (iodinated ethyl esters of fatty acids obtained from poppyseed oil; MIRIPLA Suspension Vehicle^®^, Sumitomo Pharma Co., Ltd., Tokyo, Japan) mixed with liquid paraffin and adjusted to have a CT value of about 450 HU. The raw data obtained from each scan were reconstructed with a slice thickness of 0.625 mm under the following four conditions: field of view (FOV) 120 mm + soft tissue kernel; FOV 120 mm + bone kernel; FOV 100 mm + soft tissue kernel; and FOV 100 mm + bone kernel.

### Subtraction method

**Fig. 2:**
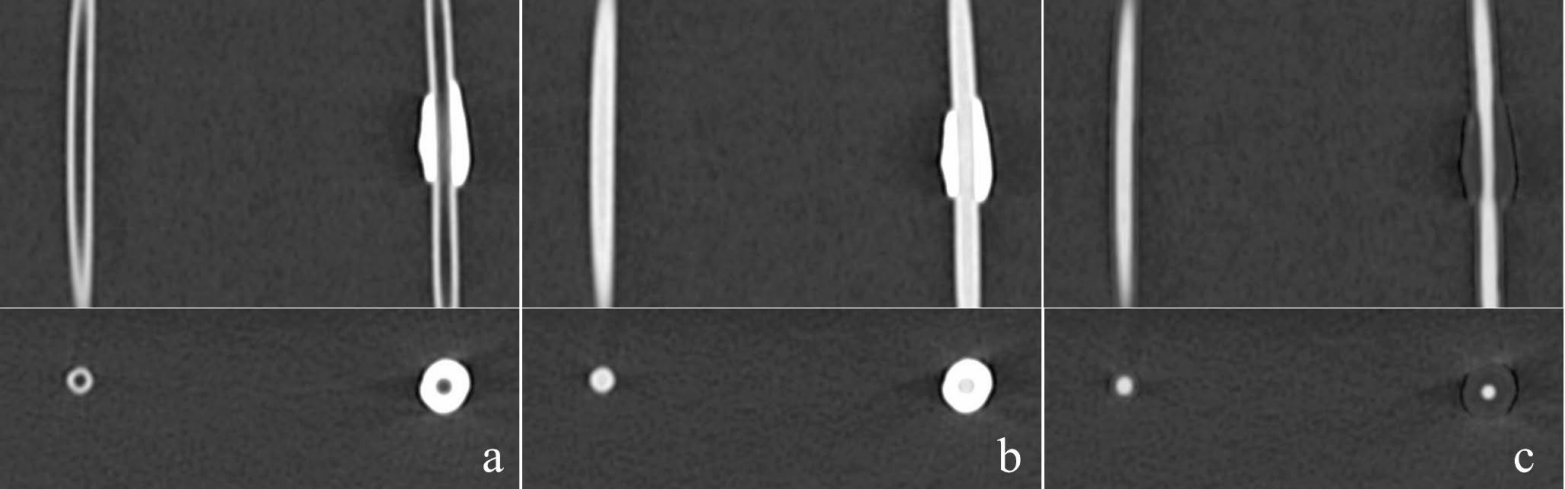
Images of simulated calcified lesions. (**a**) Image obtained at the time of saline injection (precontrast). (**b**) Image obtained at the time of contrast injection (postcontrast). (**c**) Calcification subtraction image created using precontrast and postcontrast images as the data set. The images below show each cross-section of a slice of the calcified area

Non-contrast (saline injection) and contrast images were used as the data set, and subtraction was performed on the images created under each of the above four conditions using the Cerebrovascular Subtraction application in the Synapse Vincent 3D image analysis system (Fujifilm Medical, Tokyo, Japan) to create images of subtracted simulated calcified lesions (Fig. 2). This application removes any CT value above a certain threshold (≥ 200 HU in this study) as the target of subtraction, leaving pixels with a difference in signal value before and after contrast (i.e., contrasted pixels). The rigid method (where the moved image is matched to the target image by transformation coefficients that include only translation and rotation elements) and the non-rigid method (where a transformation vector is created to move each element of the transformed image to the corresponding pixel position in the target image, and the image is transformed to match the target image) are used in this application for image registration.

### Comparison of lumen diameter

**Fig. 3:**
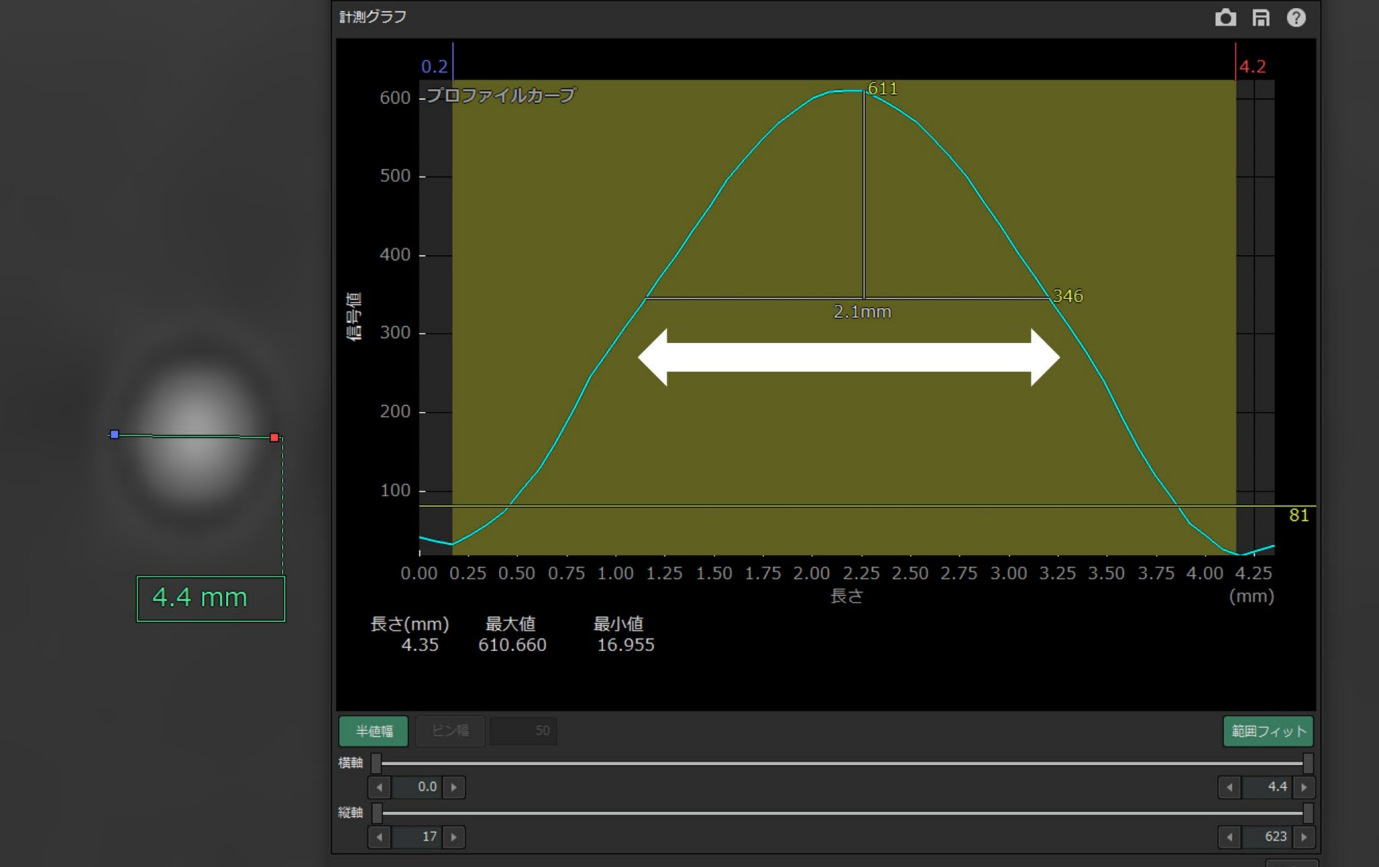
Profile curve of CT values. A straight line was placed through the center of the simulated postsubtraction vessel lumen on the cross-sectional image, and the lumen diameter was defined as the half-width (double-headed arrow) in the range indicated above 0 HU on the profile curve

The lumen diameter of the simulated vessel was measured on the post-subtraction image. Using this method, the Vincent analysis system creates a profile curve of CT values on a specified straight line and automatically measures the half-width in any range on this curve. Using this function, a straight line was placed through the center of the lumen of a simulated post-subtraction vessel, and the lumen diameter was defined as the half-width in the range indicated above 0 HU on the profile curve (Fig. 3). The lumen diameters of two simulated vessels (one with calcified lesions and the other without) in the same six slices were measured and the mean value was defined as its lumen diameter. The lumen diameter under each of the four conditions was compared. To evaluate the intra-observer reliability, the first observer performed the same measurement again after a 2-week washout period. The intra-observer reliability was assessed in terms of intraclass correlation coefficients (ICCs) and 95% confidence intervals (CIs) using a two-way mixed effects model with absolute agreement for a single measurement. ICCs were also calculated to evaluate the inter-observer reliability between the two observers, using a two-way random effects model with absolute agreement for a single rater. Four levels of reliability were defined based on the classification of ICC values proposed in [8]: poor, < 0.50 moderate, 0.50–0.74; good, 0.75–0.89; excellent, ≥ 0.90. All analyses were performed using SPSS Statistics ver. 26 (SPSS software, Armonk, NY).

### Postmortem coronary CT angiography subtraction

Two cases with highly calcified coronary arteries on postmortem CT images (Agatston calcification scores [9] of 1500 and 3570) were included. In both cases, the forensic autopsy was performed at our university with the approval of our ethics committees (approval no. 2987). After the heart was removed, catheters were inserted into the right and left coronary arteries. A Supria Grande 64-row multislice CT scanner was used to obtain precontrast and postcontrast images using a previously reported postmortem coronary angiography method [5] in which a pressurized bag is used to inject contrast medium into the removed heart. When acquiring the precontrast images, saline was injected into the coronary arteries using an infusion solution bag through a catheter, and intravascular gas was removed. A three-way stopcock was connected between the tube for injecting saline and the tube for injecting contrast medium, making it possible to switch between the injections into the catheter without moving the heart. The post-contrast images were acquired at the same position as those recorded during the pre-contrast image scanning. Additionally, the two imaging trajectories before and after administration of contrast were synchronized using the orbitally-synchronized scan function of the Supria Grande scanning system. The conditions for the two scans were the same as in the preliminary experiment. Previous reports have shown that water-soluble contrast medium leaks out of the myocardial interstitium, whereas oil-based contrast medium remain inside the vessel [10, 11]. To ensure visualization of the vessels for successful subtraction, we used the same oil-based contrast medium that was used in the preliminary experiment. In addition, the angiography method used in the present study is to inject the contrast medium for approximately 3 min (approximately 15 mL each in the left and right coronary arteries) and then perform the scan while maintaining the injection. This method can be used to visualize the peripheral vessels as well as the main coronary artery branches.

Reconstructed images were then created for the left and right coronary arteries using a slice thickness of 0.625 mm, the bone reconstruction kernel, and an FOV narrowed to approximately 100 mm. The Supria Grande also performs reconstruction by “iterative approximation processing based on a statistical model (Intelli IP),” which is expected to reduce artifacts and noise and improve image quality.

These precontrast and postcontrast reconstructed images were used as the data set for removal of calcification using the “Cerebrovascular Subtraction” application in the Vincent software, which was also used in the preliminary experiment. Three-dimensional and curved planar reconstruction images were created. Calcified areas were identified on the presubtraction images and stenotic or occlusive lesions on the postsubtraction images. For areas with severe calcification, including suspected stenotic or occlusive lesions, we compared the presubtraction and postsubtraction images of the luminal cross-section of the vessel with the gross findings for the decalcified coronary artery section.

## Results

### Preliminary experiment

**Fig. 4:**
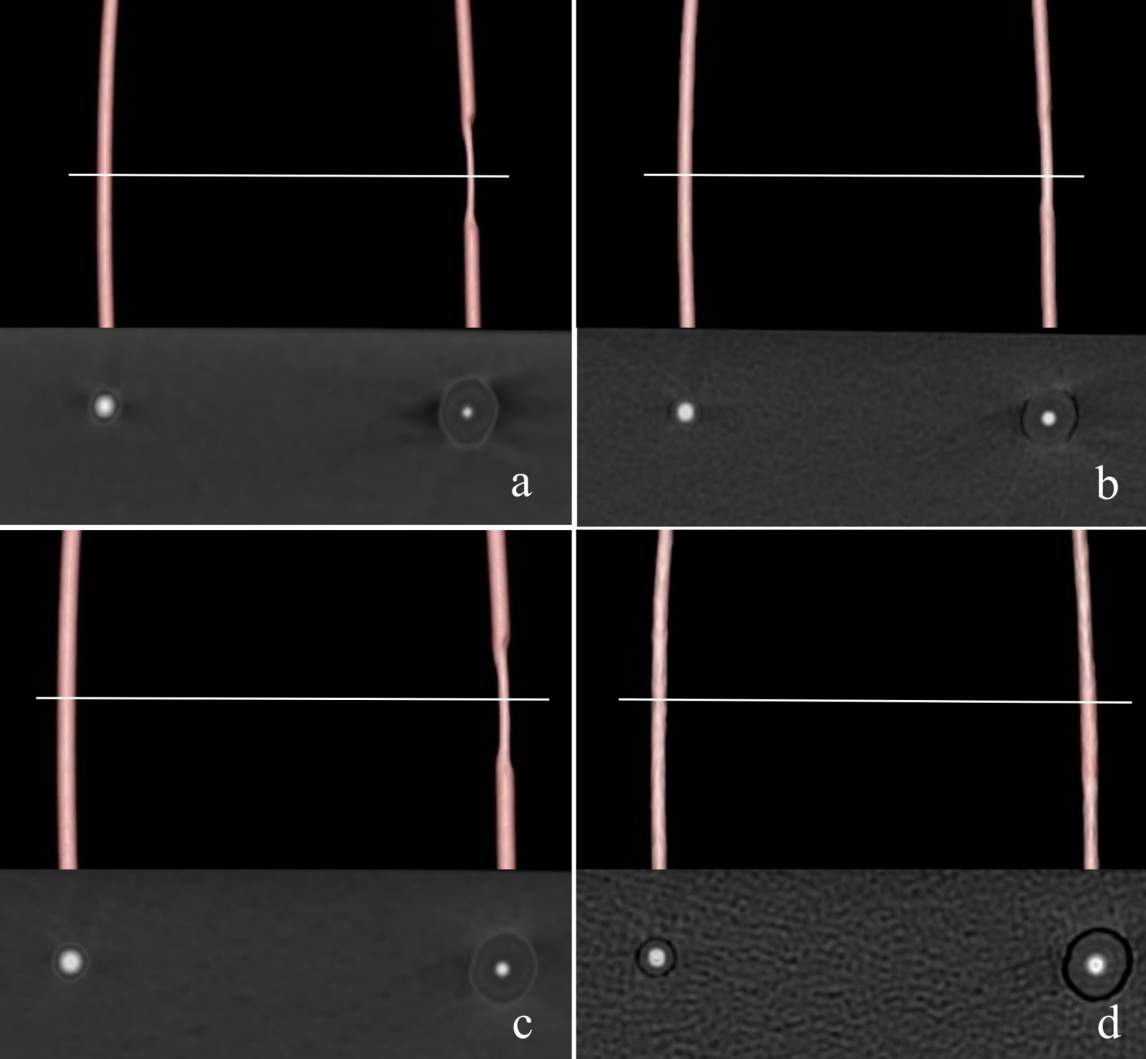
Simulated vessel images after subtraction under four conditions. (**a**) FOV 120 mm + soft tissue kernel. (**b**) FOV 120 mm + bone kernel. (**c**) FOV 100 mm + soft tissue kernel. (**d**) FOV 100 mm + bone kernel. Corresponding to the simulated calcified areas, cross-sectional views in the horizontal bar of each three-dimensional image are shown in the images below. The lumen diameter of the calcified area is narrower than that of the non-calcified area, except for (**d**) FOV 100 mm + bone kernel. FOV, field of view

The lumen diameters of the two simulated vessels after subtraction under each of the four conditions are shown in Fig. 4; Table 1. Although the diameter varied depending on the FOV and the kernel, the diameter of the noncalcified area approximated the inner diameter of the actual tube (2.2 mm) under all conditions. The diameter of the calcified area was smaller than that of the non-calcified area in the following three conditions: FOV 120 mm + soft tissue kernel; FOV 120 mm + bone kernel; and FOV 100 mm + soft tissue kernel. The greater difference was observed under the FOV 120 mm + soft tissue kernel and FOV 100 mm + soft tissue kernel conditions (0.88 mm and 0.98 mm, respectively). Under the FOV 100 mm + bone kernel condition, there was little difference in lumen diameter (0.1 mm). The intraclass correlation coefficient for the intra-observer agreement was excellent and the inter-observer agreement was good (ICC = 0.968, 95% CI [0.943, 0.982], ICC = 0.899, 95% CI [0.204, 0.971], respectively)

**Table 1:** Lumen diameters for the two simulated vessels after subtraction under four different reconstruction conditions

Reconstruction condition	FOV 120 mm + soft tissue kernel	FOV 120 mm + bone kernel	FOV 100 mm + soft tissue kernel	FOV 100 mm + bone kernel
Lumen diameter (non-calcified area) (mean ± SD mm)	2.13 ± 0.08	2.26 ± 0.05	2.4 ± 0.06	1.95 ± 0.05
Lumen diameter (calcified area) (mean ± SD mm)	1.25 ± 0.08	1.87 ± 0.05	1.42 ± 0.07	2.02 ± 0.08
Difference in lumen diameter (mean ± SD mm)	0.88 ± 0.15	0.4 ± 0.09	0.98 ± 0.1	0.1 ± 0

FOV, field of view; SD, standard deviation

### Representative examples of postmortem coronary CT angiography subtraction

#### Case 1

**Fig. 5:**
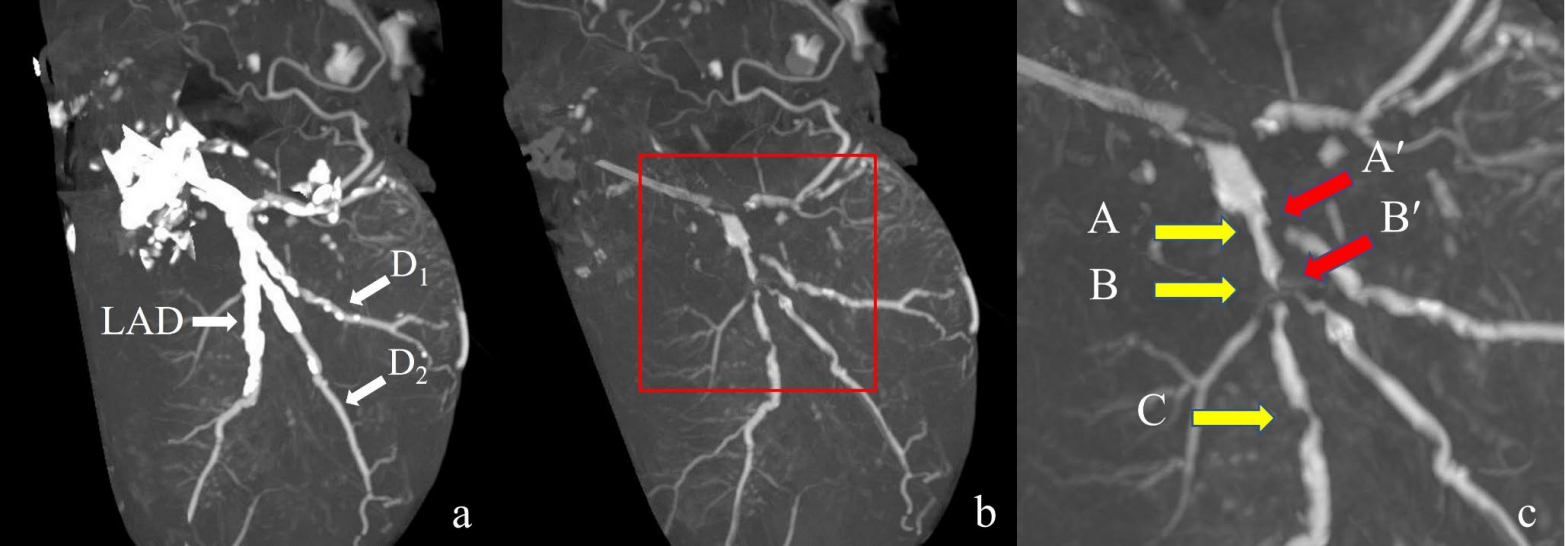
Three-dimensional image in Case 1. (**a**) A presubtraction image showing extensive calcification of the LAD. (**b**) A post-subtraction image showing a contrast defect in the proximal part of the first diagonal branch (D_1_) and patency of the LAD nearby, a contrast defect in the proximal part of the second diagonal branch (D_2_) and severe stenosis of the LAD nearby, and severe stenosis of the LAD further distal to the D_2_. (**c**) Enlarged image within the red square of the image in (**b**). The capital letters with arrows correspond to each image shown in Fig. 6. LAD, left anterior descending branch

**Fig. 6:**
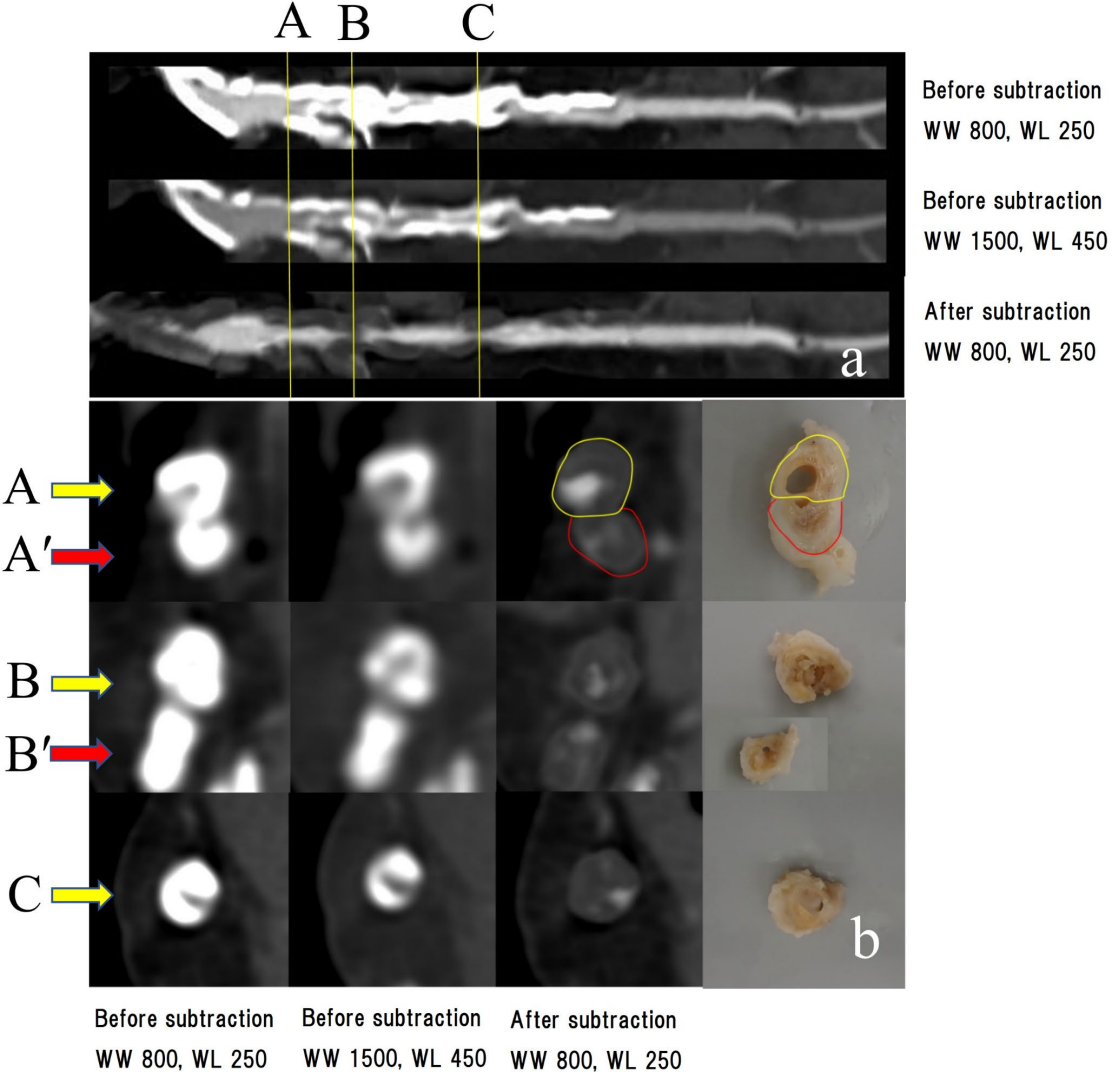
Coronary artery findings in Case 1. (**a**) Curved planar reconstruction images. Top row: image before subtraction (WW 800, WL 250). Middle row: image before subtraction (WW 1500, WL 450). Bottom row: image after subtraction (WW 800, WL 250). Cross-sectional views are shown in vertical lines in (**b**). (**b**) Cross-sectional views of each site. From left to right: before subtraction (WW 800, WL 250), before subtraction (WW 1500, WL 450), after subtraction (WW 800, WL 250), and gross findings. Before subtraction, it was difficult to evaluate the presence of occlusion or degree of stenosis under any of the reading conditions owing to high calcification; after subtraction, patency and severe stenosis or occlusion were confirmed. The degree of stenosis and the morphology in each lesion were similar to the gross findings WW, window wide; WL, window level

**Fig. 7:**
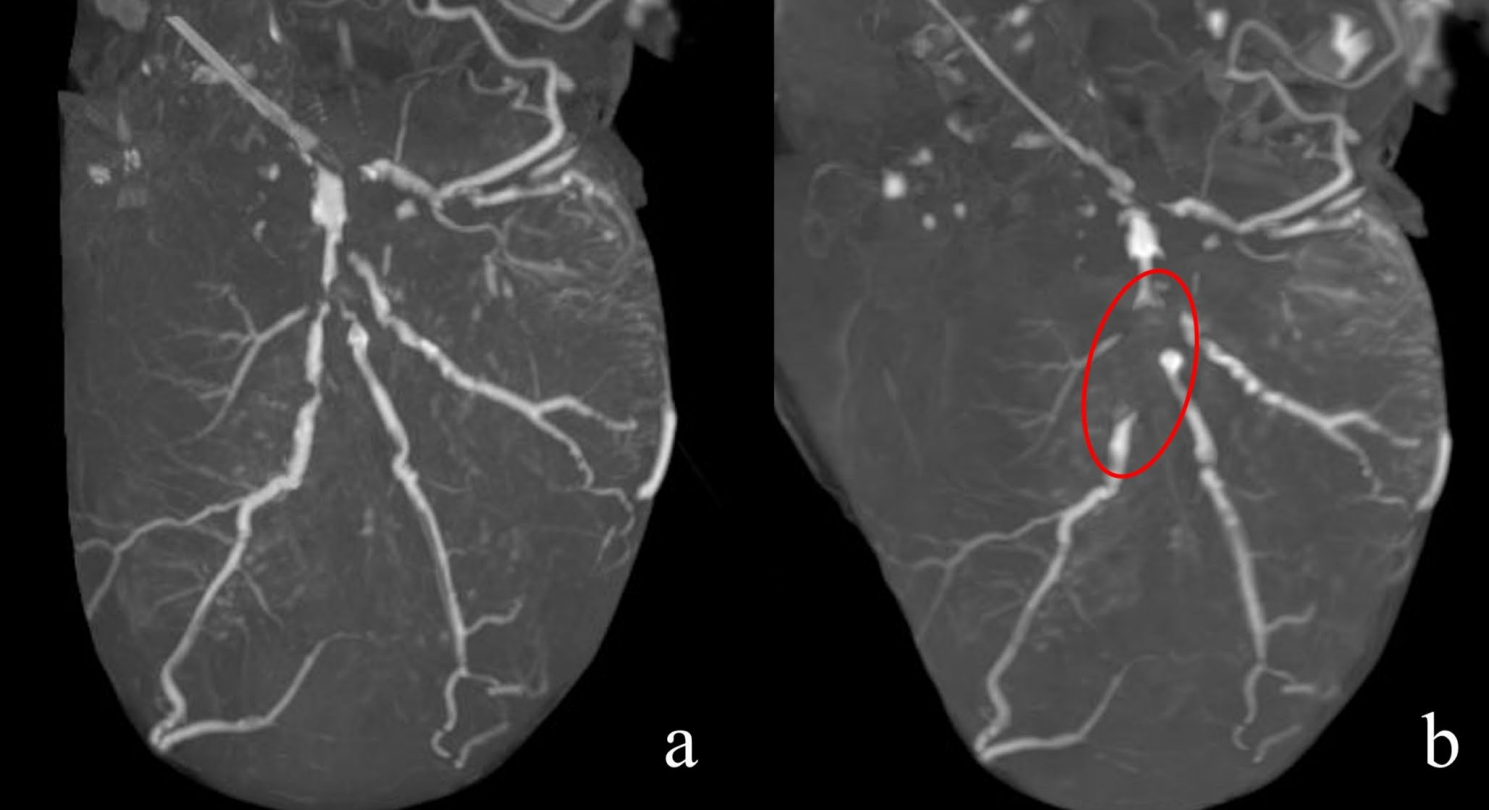
Comparison of subtracted images under different reconstruction conditions in Case 1. (**a**) FOV 100 mm + bone kernel. (**b**) FOV 120 mm + soft tissue kernel. The calcification areas of the LAD (red circle in **b**) are over-subtracted under the condition of FOV 120 mm + soft tissue kernel

A three-dimensional image acquired using the maximum intensity projection method is shown in Fig. 5. The presubtraction image shows extensive calcification of the left anterior descending branch (LAD). The postsubtraction image shows a contrast defect in the proximal part of the first diagonal branch (D_1_) and the patency of the LAD nearby, a contrast defect in the proximal part of the second diagonal branch (D_2_) and severe stenosis of the LAD nearby, and severe stenosis of the LAD further distal to the D_2_. Curved planar reconstruction images and cross-sectional images of the respective sections in the LAD are shown in Fig. 6. Before subtraction, it was difficult to evaluate the presence of occlusion or the degree of stenosis under any of the reading conditions (WW [window wide] 800, WL [window level] 250, WW 1500, and WL 450) because of high calcification. After subtraction, patency and stenosis or occlusion were examined. The degree of stenosis and its morphology in each lesion were similar to the gross findings. The defect from the main trunk to the proximal part of the circumferential branch was caused by misregistration due to over-insertion of the catheter in the precontrast image. Figure 7 shows a comparison of two subtracted images under different reconstruction conditions. The calcification areas are over-subtracted on the condition of FOV 120 mm + soft tissue kernel compared with FOV 100 mm + bone kernel.

### Case 2

**Fig. 8:**
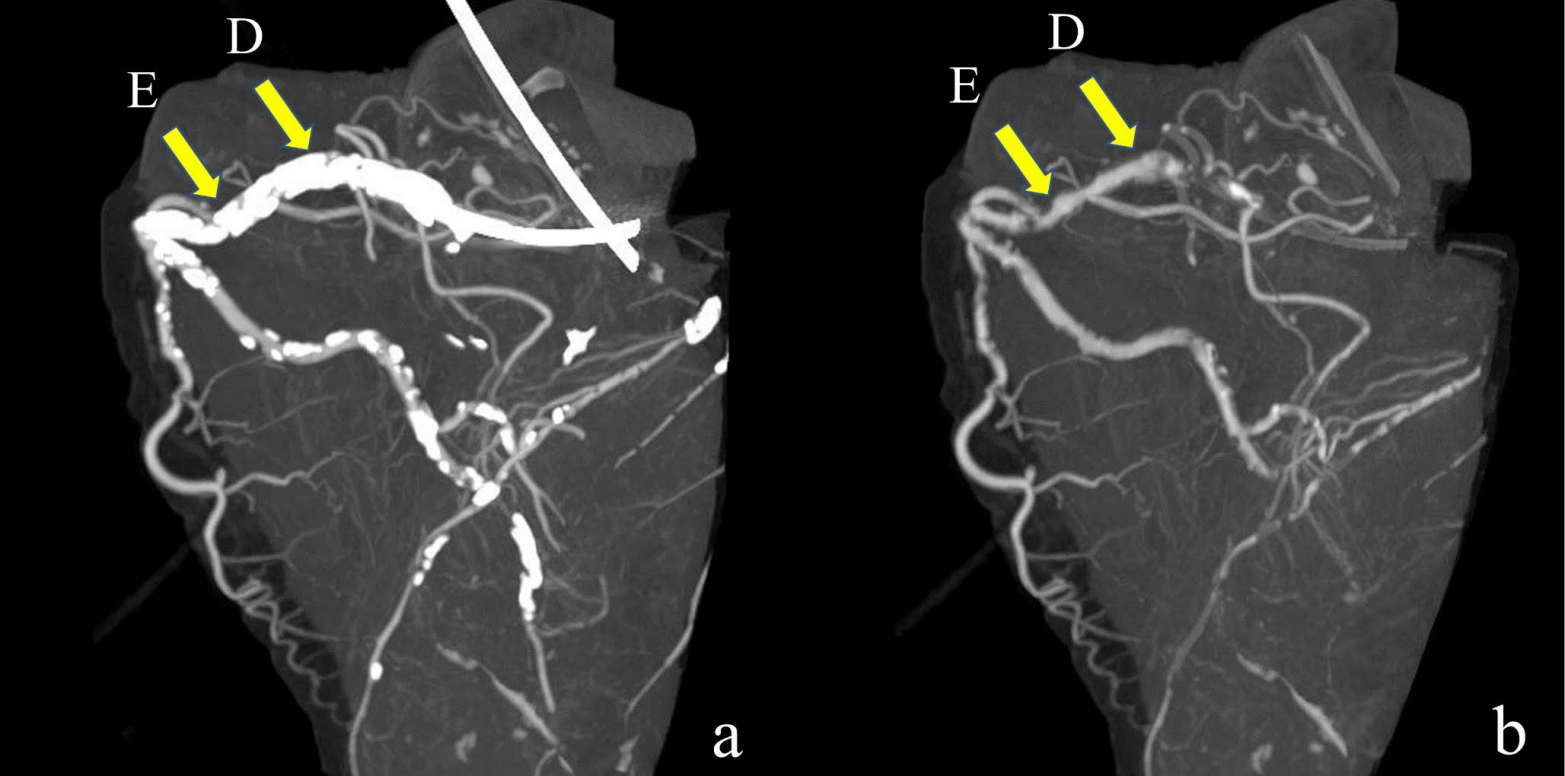
Three-dimensional image in Case 2. (**a**) Presubtraction image showing extensive calcification of the proximal right coronary artery. (**a**) Postsubtraction image showing lumen patency in the area where calcification is present. The capital letters with arrows correspond to each image in Fig. 9

**Fig. 9:**
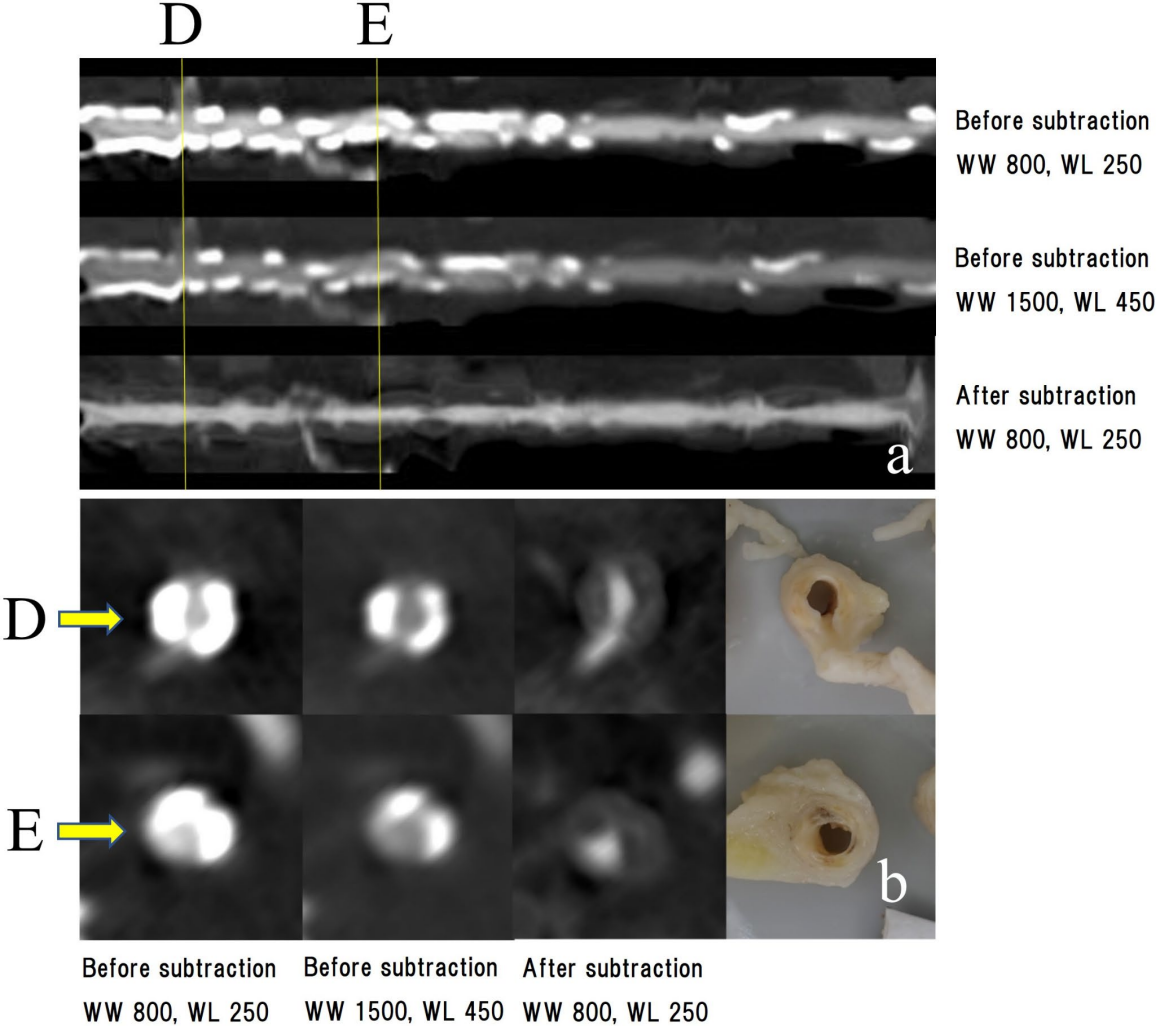
Coronary artery findings in Case 2. (**a**) Curved planar reconstruction images. Top row: image before subtraction (WW800, WL250), middle row: image before subtraction (WW 1500, WL 450), and bottom row: image after subtraction (WW 800, WL 250). Cross-sectional views in vertical lines are shown in (**b**). (**b**) Cross-sectional views of each site. From left to right: before subtraction (WW 800, WL 250), before subtraction (WW 1500, WL 450), after subtraction (WW 800, WL 250), and gross findings. Before subtraction, the lumen is open under both reading conditions, but the degree of stenosis appears to vary depending on WW and WL. After subtraction, the degree and morphology of the stenosis in the images and gross findings are similar WL, window level; WW, window wide

A three-dimensional image obtained using the maximum intensity projection method is shown in Fig. 8. Presubtraction images showed extensive calcification in the proximal right coronary artery. A curved planar reconstruction image and two cross-sectional images are shown in Fig. 9. Before subtraction, the patency of the lumen was determined under both reading conditions, and the degree of stenosis appeared to vary depending on WW and WL. After subtraction, the degree and morphology of the stenosis in the images and gross findings were similar.

## Discussion

Calcified lesions can be a major obstacle to assessment of stenosis by coronary CT angiography both ante- and postmortem because blooming and partial volume artifacts make it difficult to observe the lumen. In clinical practice, an Agatston calcium score of > 400 for a target coronary artery is associated with a significant decrease in specificity [12], and in CORE 64, a multicenter study of the diagnostic performance of coronary angiography by 64-row multidetector CT, cases with a calcium score of > 600 were excluded [13]. The impact of coronary calcium on the decision to perform diagnostic contrast CT in symptomatic patients with a calcium score of > 400 has been reported to be “uncertain” [14]. There have been no studies of the performance of contrast-enhanced CT in diagnosis of coronary artery lesions in cases with high calcification in the forensic field; however, it is believed that this imaging method overestimates stenosis, as in the clinical setting [15].

A two-scan subtraction method has been proposed to overcome the problem of calcified lesions [1–7]. Theoretically, the subtraction technique might be able to remove calcification in contrast-enhanced data sets by subtracting the precontrast CT imaging data but requires exact alignment. Therefore, in clinical practice, subtraction requires special imaging to minimize motion artifact in the heart and registration to allow accurate alignment. The coronary CT subtraction studies performed in the clinical field have used specialized CT scanners and consoles with specialized subtraction algorithms. However, an international multicenter study that used invasive coronary angiography as a reference reported a high misregistration rate, although the method reduced the false positivity rate from 72 to 33% [7]. Some researchers attribute this misregistration to beating of the heart and believe that the two-scan subtraction method has limitations because of this artifact [7, 16]. However, given that the heart does not beat after death, this method may be more effective in postmortem CT angiography, which is why we conducted this study.

We performed a preliminary experiment to determine the optimal image acquisition conditions for subtraction. Accurate registration of structures is essential when using the subtraction method because even small misalignments can produce striking and disruptive bright and dark artifacts [17]. For more accurate registration, it is presumed that it is beneficial to use images with a higher spatial resolution in which the edges of the target material are more clearly defined. Therefore, we created reconstructed images under four FOV and reconstruction kernel conditions, which affect spatial resolution, and then performed subtraction and compared each image. We found that the most accurate subtraction was performed using reconstructed images with a narrower FOV and the bone kernel, which provided higher spatial resolution. This was also demonstrated in a comparison using actual specimens.

On the other hand, even in postmortem angiography, the presence of gas in the coronary arteries and slight misalignment of the scan positions between two scans can interfere with registration. To minimize these problems, we infused saline to remove intravascular gas during acquisition of precontrast images and used the orbitally synchronized scan function of the Supria Grande to synchronize the positional information of the precontrast and postcontrast scans.

In two cases in which these conditions were set, the postmortem CT angiography subtraction method allowed evaluation of lumen patency and the degree of stenosis in contrast-enhanced images, where lumen evaluation had been difficult because of high calcification. The findings were morphologically similar to the macroscopic findings. Although the subtraction application in the Vincent software is for cerebrovascular vessels and not specifically for coronary vessels, good images were obtained because there is no interference from the movement of the heart, and the presence of morphological features such as calcified lesions and the heart itself, and because the vessels were well visualized by the postmortem coronary angiography method using oil-based contrast medium. The main limitation of this validation study is that it was performed using specific software. The process of image registration involves not only uncertainties in the input data (e.g., artifacts), but also general uncertainties in the software, errors due to fusion algorithms, and uncertainties resulting from specific registration results. In clinical practice, there is no standardized formula to express this uncertainty [18]. Given that the various types of software use different algorithms for subtraction, further studies are needed to determine whether our method is feasible using other types of software.

In recent years, postmortem CT angiography has been performed using minimally invasive whole-body angiography rather than angiography of removed organs [19, 20]. However, we believe that the concept and methods used in our study can be applied to whole-body angiography. In the future, we plan to add radiological evaluation (assessment of image quality by a radiologist) and histopathological evaluation (measurement of luminal area) to validate the diagnostic performance of this subtraction method in postmortem examinations in a larger number of cases.
